# Abstracts of the 2024 Canadian Association of Medical Oncologists Annual Scientific Meeting

**DOI:** 10.3390/curroncol32020105

**Published:** 2025-02-12

**Authors:** Alexi Campbell, Sharlene Gill, Desirée Hao, Suneil Khanna, Erin Powell, Moira Rushton, Maryam Soleimani, Stephen Welch

**Affiliations:** Canadian Association of Medical Oncologists, Ottawa, ON K1Z 1A2, Canada

**Keywords:** medical oncology, cancer, research

## Abstract

On behalf of the Canadian Association of Medical Oncologists, we are pleased to present the abstracts of the 2024 Annual Scientific Meeting. The CAMO Annual Scientific Meeting (ASM) took place from 24–26 October 2024 in an in-person event in Ottawa, ON. Twenty-four (24) abstracts were selected for presentation as oral presentations and poster presentations. Awards for the top three (3) abstracts were presented during the ASM; they have been marked as “Award Recipient”. We congratulate all presenters on their research work and contribution.

## 1. Oral Presentation|01_CAMO_2024

Award Recipient

**Real-World Practice Patterns, Treatment-Related Toxicity, and Survival Outcomes in Older Patients with Metastatic Renal Cell Carcinoma: Results From The Canadian Kidney Cancer Information System (CKCis) ^†^**

Lauren Curry ^1^, Sunita Ghosh ^2^, Erica Arenovich ^3^, Simon Tanguay ^4^, Aly-Khan A. Lalani ^5^, Daniel Yick Chin Heng ^6^, Bimal Bhindi ^7^, Naveen S. Basappa ^8^, Jeffrey Graham ^9^, Georg A. Bjarnason ^10^, Rodney H Breau ^11^, Vincent Castonguay ^12^, Denis Soulieres ^13^, Frederic Pouliot ^14^, Dominick Bosse ^15^, Christian K. Kollmannsberger ^1^, Antonio Finelli ^16^, Nazanin Fallah-rad ^16^ and Maryam Soleimani ^1,^*

1. British Columbia Cancer–Vancouver Cancer Centre, Vancouver, BC, Canada
2. University of Alberta, Edmonton, AB, Canada
3. QEII Health Sciences Centre, Halifax, NS, Canada
4. McGill University Health Center, Montreal, QC, Canada
5. Department of Oncology, Juravinski Cancer Centre, McMaster University, Hamilton, ON, Canada
6. Tom Baker Cancer Centre, University of Calgary, Calgary, AB, Canada
7. Southern Alberta Institute of Urology, Calgary, AB, Canada
8. Cross Cancer Institute, University of Alberta, Edmonton, AB, Canada
9. University of Manitoba, Winnipeg, MB, Canada
10. Sunnybrook Odette Cancer Centre, Toronto, ON, Canada
11. Ottawa Hospital Research Institute, Ottawa, ON, Canada
12. Medical Oncology, Centre Hospitalier Universitaire de Québec, Université Laval, Quebec City, QC, Canada
13. Centre Hospitalier de l’Université de Montréal, Montreal, QC, Canada
14. CHU de Québec—Université Laval, Québec, QC, Canada
15. University of Ottawa, Ottawa, ON, Canada
16. Princess Margaret Cancer Centre, University Health Network, Toronto, ON, Canada

^†^  The abstract had been accepted for 2024 ASCO Genitourinary Cancers Symposium. Details: *Journal of Clinical Oncology*, 29 January 2024, Volume 42, number 4 supplemental, https://ascopubs.org/doi/10.1200/JCO.2024.42.4_suppl.366.

**Objectives**

There is a paucity of data with respect to optimal management of metastatic renal cell carcinoma (mRCC) in older adults. Real-world data may help close this knowledge gap and improve care for older patients with mRCC.

**Methods**

The Canadian Kidney Cancer information system (CKCis) was utilized to identify patients with mRCC, categorizing them as either older (age ≥ 75 years) or younger (age < 75 years). We compared 1L mRCC management strategies and treatment-related toxicities. Secondary outcomes were OS and TTD.

**Results**

A total of 2576 patients were included (*n* = 2203 <75 years old; *n* = 373 ≥75 years old). Older patients had more comorbidities (5+, 95% vs. 67%, *p* < 0.0001) and more frequently had KPS < 70% (18% vs. 13%, *p* = 0.01). Older patients underwent metastasectomy less frequently (15% vs. 25%, *p* = 0.0001) and were less likely to be enrolled in clinical trials (10% vs. 24%, *p* < 0.0001). Older patients received 1L TKI monotherapy more frequently (79% vs. 69%, *p* < 0.0001) than ICI-based treatment, even when adjusted by year to account for changes in practice patterns in the post-ICI era (65% vs. 44%, *p* < 0.0001). There was no difference in the type of ICI regimen prescribed when compared by age (*p* = 0.61). Older patients did not experience more frequent treatment-related toxicities with ICI-based treatment. They did however experience more grade 3+ toxicity with TKI monotherapy. Older patients had shorter OS even when controlling for IMDC score, CCI and histology (HR 1.21, 95%CI 1.03–1.43, *p* = 0.02). There was no difference in TTD by groups.

**Conclusions**

Patients ≥ 75 years of age received TKI monotherapy more frequently than those < 75 years of age, though when they received ICI-based regimens, they did not experience more treatment-related toxicities. Clinicians should individualize treatments for older patients not solely based on age, but after discussion of all available options in a patient-centered manner, considering comorbidities, disease burden, and patient preferences.

## 2. Oral Presentation|02_CAMO_2024

**Impact of Concurrent Antibiotics On Survival with Immunotherapy in the CCTG CO.26 and PA.7 Trials of Metastatic Colorectal and Pancreatic Cancer ^†^**

Cynthia Yeung ^1^, Emma Titmuss ^1^, Dongsheng Tu ^2^, Daniel Renouf ^1^, Sharlene Gill ^1^, Jennifer J Knox ^3^, Eric Chen ^3^, Chris J O’Callaghan ^2^, Jonathan M Loree ^1^

1. BC Cancer, Vancouver, BC, Canada
2. Canadian Cancer Trials Group, Queen’s University, Kingston, ON, Canada
3. Princess Margaret Cancer Centre, UHN, University of Toronto, Toronto, ON, Canada

^†^  This work has been published as an abstract for 2024 ASCO Annual Meeting I. 29 May 2024, *Journal of Clinical Oncology*, Volume 42, Number 16_suppl, https://doi.org/10.1200/JCO.2024.42.16_suppl.2609.

**Background**

The interplay between antibiotics and the microbiome is hypothesized to affect immune checkpoint inhibitor (ICI) efficacy, and antibiotic administration is common in patients with cancer. Existing literature suggests that antibiotics are associated with worse ICI outcomes, particularly in lung cancer and melanoma.

**Methods**

We reviewed concurrent antibiotics received in two randomized phase II Canadian Cancer Trials Group (CCTG) clinical trials. CO.26 evaluated durvalumab and tremelimumab versus best supportive care only (BSC) in metastatic refractory colorectal cancer (CRC). PA.7 evaluated chemotherapy with/without durvalumab and tremelimumab in metastatic pancreatic cancer.

**Result**

In CO.26 (*n* = 180), concurrent antibiotic exposure was associated with improved overall survival (OS) (7.4 vs. 6.2 months, hazard ratio (HR) 0.57 (95% confidence interval (CI) 0.38–0.86), *p* = 0.007) in patients who received ICIs, but not in those receiving BSC (3.5 vs. 4.5 months, HR 1.49 (95%CI 0.81–2.73), *p* = 0.20); (p-interaction = 0.094). Concurrent exposure to ICIs and fluoroquinolones (9.4 vs. 6.2 months, aHR 0.50 (95%CI 0.30–0.82), *p* = 0.0067), but not penicillins or cephalosporins, was associated with better OS.

In PA.7 (*n* = 180), antibiotics and fluoroquinolones were not associated with OS in patients who did/did not receive ICIs (Antibiotics: 9.7 vs. 10.8 months, HR 0.97 (95%CI 0.64, 1.46), *p* = 0.87; 7.4 vs. 10.3 months, HR 1.22 (95%CI 0.72, 2.09), *p* = 0.46, respectively. There was no impact on ICI efficacy when analysis was limited to fluoroquinolones in PA.7: HR 0.74 (95%CI 0.47, 1.15), *p* = 0.18.

**Conclusions**

Concurrent antibiotic exposure, specifically fluoroquinolones, with ICIs was associated with improved OS in metastatic CRC. This is discrepant from prior reports in other histologies and suggests the microbiome may impact ICI efficacy uniquely in CRC.

## 3. Oral Presentation|03_CAMO_2024

**Implementation of a Hepatitis B Screening Program in Patients Receiving Systemic Anti-Cancer Therapy: A Canadian Single Center Retrospective Review ^†^**

Jennifer Leigh ^1^, Ranjeeta Mallick ^2^, Stephanie Brule ^1^, Lisa Rambout ^3^, Jennifer Newton ^4^, Dominick Bossé ^1^, Sara Moore ^1^ and Joanna Gotfrit ^1^

1. Division of Medical Oncology, Department of Medicine, The Ottawa Hospital Ottawa, ON, Canada
2. Ottawa Hospital Research Institute, Ottawa, ON, Canada
3. The Ottawa Hospital Pharmacy Department, Ottawa, ON, Canada
4. The Ottawa Hospital Cancer Center, Ottawa, ON, Canada

^†^  The full article was published by current oncology themselves on 30 December 2024. Here is the DOI: https://doi.org/10.3390/curroncol32010020.

**Objective**

To assess the success of the real-world implementation of a hepatitis B (HBV) screening pilot program and to ascertain whether an institutional HBV screening policy leads to screening in all eligible patients.

**Methods**

The Ottawa Hospital Cancer Centre implemented a pilot HBV screening program for patients receiving FOLFOX-based regimens or FOLFOXIRI between January and April 2023. The program incorporated screening tests into treatment plans and specified pharmacy and nursing pre-treatment checks. Following the pilot, charts were retrospectively reviewed. The primary endpoint was to identify the proportion of patients who underwent screening. Univariate analyses assessed the association between baseline characteristics and failure to screen. We also reviewed quality metrics including time to testing and result, number of duplicate screens, and treatment delays.

**Results**

There were 32/42 eligible patients (76.2%) who completed screening and 5(11.9%) had a positive screen. Median age was 64 years, with 57.1% male, and 71.4% with colorectal cancer. Of those screened, the majority (59.5%) completed screening prior to first treatment as intended; however, 38.1% were drawn on the day of treatment. A total of 9.52% of patients had duplicate screening inadvertently. Four patients who tested positive were referred to Infectious Diseases. There were no treatment delays due to pending screening and no HBV reactivation. Receipt of prior systemic therapy was significantly associated with failure to screen (55% vs. 95%, OR 17.1 (95%CI 1.92–153), *p* = 0.011). Factors including age, sex, primary disease site, therapy intent, and regimen received were not significantly associated with screening completion or HBV positivity.

**Conclusions**

The results of this pilot highlight the importance of building HBV screening into standardized treatment plans and engaging all team members to ensure high levels of screening. Prior systemic therapy receipt was associated with failure to screen, and thus programs should include education on the necessity of screening as recommended by medical guidelines.

## 4. Oral Presentation|04_CAMO_2024

**Improving Dihydropyrimidine Dehydrogenase Genotyping (DPYD) Prior To Initiation of Fluoropyrimidine-Based Chemotherapy at a Community-Based Hospital: A Quality Improvement Initiative ^†^**

Coralea Kappel ^1,‡^, David Nguyen ^1,‡^, Mary Yousef ^2^, Monica Panetta ^2^, Coralea Kappel ^1^, Carlos Muzlera ^1^, Mathew Hall ^1^, Mitchell Elliott ^1^, Heather Bussey ^2^, Tiffany Casalinuovo ^2^, Mary Mahler ^1,2^ and Charles Lim ^1,2^

1. University of Toronto, Division of Medical Oncology, Toronto, ON, Canada
2. Carlo Fidani Regional Cancer Centre, Trillium Health Partners-Credit Valley Hospital, Mississauga, ON, Canada

^†^  only abstract was previously published. *JCO Oncology Practice*, Volume 20, Number 10_suppl, https://doi.org/10.1200/OP.2024.20.10_suppl.347.
^‡^  denotes co-first author.

**Background and Rationale**

Dihydropyrimidine dehydrogenase gene (DPYD) testing is crucial in preventing toxicities from fluoropyrimidine chemotherapy. Despite benefits and established guidelines, its adoption within healthcare organizations remains inconsistent and suboptimal. We implemented a quality improvement project to improve DPYD testing prior to fluoropyrimidine chemotherapy at a community-based hospital.

**Aims**

We aimed to increase DPYD testing for patients receiving fluoropyrimidine-based chemotherapy from 65% to 95% between July 2023 to July 2024. Balancing measures included DPYD testing turnaround time.

**Methods**

For baseline diagnostics, 248 patients were identified who received fluoropyrimidine-based chemotherapy between 1 May 2023 to 4 August 2023. We performed manual chart review on 126 patients and identified frequency of DPD testing, frequency of variant DPYD results, turn-around time for DPYD testing. Patients were excluded if they received fluoropyrimidine-based chemotherapy prior to 1 May 2023.

A multidisciplinary team was made including medical oncology residents and staff, pharmacists, nurses, and clinical informatics specialists to identify and address barriers to implementation. Barriers identified included lack of established workflow, awareness and importance of testing. A rapid cycle improvement approach was used to optimize DPYD testing for patients prior to receiving fluoropyrimidine chemotherapy. The team used Plan-Do-Study-Act (PDSA) cycles to address barriers. A p-chart was used to analyze the results.

**Results**

PDSA cycles included education sessions to breast medical oncologists and medical oncology nurses, and development and implementation of a best-practice advisory in the electronic medical record. The percentage of eligible patients receiving DPYD testing increased from 65% to 91% in the total population. For breast cancer patients, the rate increased from 16.7% to 100%. For gastrointestinal cancer patients, the rate increased from 56.1% to 88%.

**Conclusions**

Through PDSA cycles, we improved the frequency of DPYD testing for patients receiving fluoropyrimidine chemotherapy from 65% to 91%. Future work will assess sustainability.

## 5. Oral Presentation|05_CAMO_2024

**Somatic Tissue Testing in Prostate Cancer Using the Oncomine V3 Panel: A Single-Institution Retrospective Analysis †**

Lilian Hanna ^1,2^, Nicole Park ^3^, Xin Wang ^1,2^, Larissa Waldman ^4^, Yael Silberman ^4^, Urban Emmenegger ^1,2^, Michelle R. Downes ^3,5,6^ and Martin Smoragiewicz ^1,2^

1. Division of Medical Oncology and Hematology, Odette Cancer Centre, Toronto, ON, Canada
2. Sunnybrook Health Sciences Centre, Toronto, ON, Canada
3. Department of Medical Oncology, University of Toronto, Toronto, ON, Canada
4. Department of Laboratory Medicine and Molecular Diagnostics, Sunnybrook Health Sciences Centre, Toronto, ON, Canada
5. Department of Molecular Genetics, University of Toronto. Cancer Genetics and High Risk Program, Sunnybrook Health Sciences Centre, Toronto, ON, Canada
6. Department of Laboratory Medicine and Pathobiology, University of Toronto. Department of Laboratory Medicine and Molecular Diagnostics, Sunnybrook Health Sciences Centre, Toronto, ON, Canada

^†^  Only the abstract has been published; please see the following link: https://ascopubs.org/doi/10.1200/JCO.2024.42.16_suppl.e17002.

**Objective**

To describe the somatic tissue testing (STT) experience for prostate cancer at Sunnybrook Health Sciences Centre using the Oncomine v3 gene panel and the utility of reporting variants beyond funded genes in Ontario (BRCA1/2, ATM, PALB2).

**Methods**

Sixty-two prostate cancer (PC) patients between 1 May 2021 and 31 January 2023 were included. Clinical characteristics were gathered via chart review. Somatic variants were extracted by an annotation specialist.

**Results**

Tests were ordered in the locoregional setting in 46.8% (*n* = 29), metastatic castrate-sensitive setting in 37.1% (*n* = 23), metastatic castrate-resistant (CR) setting in 8.1% (*n* = 5) and non-metastatic CR setting in 3.2% (*n* = 2). Tests were ordered by reflex testing in 64.5% (*n* = 40), Medical Oncology in 16% (*n* = 10), Radiation Oncology in 13% (*n* = 8) and others in 6.5% (*n* = 4). Fifty samples (80.6%) were of prostatic origin vs. 12 (19.4%) of metastatic sites. The median test turn-around time was 28.5 days (range 5–57). There were 63 tier 1 and 2 variants, listed in Table 1. Eight of 63 variants (12.7%) were within funded genes whereas 55/63 (87.3%) were within unfunded genes. CDK12/FANCA/NBN (*n* = 7, 11.2%) are investigated in poly (ADP-ribose) polymerase inhibitor trials and PMS2 deficiency (*n* = 1, 1.6%) can select for immune checkpoint inhibitors. PIK3R1/PIK3CA/AKT1-mutated tumors (*n* = 10, 16.1%) have shown response to AKT-inhibitors. SPOP-mutated tumors (*n* = 6, 9.7%) are associated with prolonged response to androgen receptor blockade whereas PTEN/TP53/RB1-altered tumors (*n* = 11, 17.7%) are associated with anti-androgen resistance and worse prognosis.

**Conclusions**

The majority of STT variants identified were within unfunded genes; many of which with prognostic and predictive value. This highlights that STT with extended gene panels early in the disease course may help in delivering personalized PC care.

## 6. Oral Presentation|06_CAMO_2024

**Asian Ancestry Is Associated with Long Term Survival in EGFR Positive Non-Small Cell Lung Cancer (NSCLC) Treated with First-Line Tyrosine Kinase Inhibitors (TKI): A Multicentre Canadian Cohort**

Heather Halperin ^1^, Amanda J.W. Gibson ^1^, Ali Hussein ^2^, Mike Sung, Pascale Tomasini ^3^, Michelle Dean ^2^, Jennifer Law ^4^, Natasha Leighl ^4^, Cheryl Ho ^3^, Vishal Navani ^2^ and Desiree Hao ^2^

1. Department of Internal Medicine, University of Calgary, Calgary, AB, Canada
2. Department of Oncology, Section of Medical Oncology, Tom Baker Cancer Centre, Calgary, AB, Canada
3. Department of Oncology, Section of Medical Oncology, BC Cancer, Vancouver, BC, Canada
4. Department of Oncology, Section of Medical Oncology, Princess Margret Hospital, Toronto, ON, Canada

**Background**

In Asian predominant populations, multiple reports identify Asian ancestry as prognostic of improved outcome in EGFRmut^+^ NSCLC populations when treated with 1st/2nd generation TKI. Generalizability to more heterogeneous populations is less understood.

**Objective**

To identify clinical characteristics associated with long-term survival (LTS) in patients from heterogenous Canadian populations with advanced NSCLC with EGFRmut^+^ treated with first-line TKIs.

**Methods**

De novo advanced EGFRmut^+^ NSCLC diagnoses receiving a 1st/2nd generation EGFR-TKI between 2004 and 2016 were included. Demographic, clinical, treatment and outcome details were extracted from three sources: two province-wide, multi-centre registries, the Alberta Glans-Look Lung Cancer Research Database (GLR), and the British Columbia Cancer Agency (BCCA), along with data from Princess Margaret Cancer Centre (PM), a single centre, urban, tertiary hospital. Survival time from TKI initiation was divided into LTS, patients surviving longer than the upper quartile (34.4 months) and the remaining patients described as ‘average term survivors’ (ATS).

**Results**

Of 577 patients (GLR: 246, PM: 112 BCCA: 219), the median overall survival was 20.3 months. From initiation of TKI, LTS median survival was 48.5 months, whereas ATS was 15.7 months. The LTS cohort differed significantly from ATS in many clinical and pathologic characteristics, including being significantly more likely to be of Asian ancestry (50% vs. 42%, *p* = 0.023), Female (74% vs. 64%, *p* = 0.037) and less likely to harbour L858R-mutation(41% vs. 66%, *p* = 0.004). In multivariate analysis, Asian ancestry (HR 1.63, *p* = 0.022) was an independent prognostic factor of longer survival times, whereas L858R mutation (HR 0.62, *p* = 0.025) was an independent prognostic factor of shorter overall survival.

**Conclusions**

This study confirmed previously known prognosticators within a real-world multicentre Canadian cohort. This included Asian ancestry, and female sex were associated with better overall survival and L858R-mutation associated with shorter survival. Future studies to better understand the impact of socioeconomic factors and biologic reasons for ancestry resulting in different prognosis are needed to better guide treatment management.

## 7. Oral Presentation|07_CAMO_2024

**Meaningful Endpoints in Patients with Advanced Melanoma Being Considered for Adjuvant Systemic Therapy**

Daniel Goldshtein ^1^ and Baskoro (Adi) Kartolo ^2^

1. Internal Medicine, McMaster University, Hamilton, ON, Canada
2. Medical Oncology, McMaster University, Hamilton, ON, Canada

**Objective**

Landmark adjuvant melanoma trials demonstrated disease-free survival (DFS) but no overall survival (OS) benefits. Given that patients’ values were rarely incorporated in clinical trial designs, we aimed to understand patients’ perception of meaningful study endpoints and magnitude of treatment benefits.

**Methods**

This prospective, online-survey study enrolled all consecutive patients at the Juravinski Cancer Centre with resected stage IIB-IV cutaneous melanoma and considered for adjuvant systemic therapy. Primary outcome was the most important perceived study endpoint. Trade-offs (e.g., side effects, quality of life) were explored in secondary outcomes.

**Results**

From May 2023 to February 2024, the survey had a completion rate of 46% (36/79). A total of 22% of patients had resected stage IIB/C melanoma. OS (42%) was the most important perceived study endpoint by our patients, followed by quality of life (28%) and DFS (28%). While 63% would consider treatment for DFS with unknown OS benefit, only 20%, 14%, and 14% remain interested in treatment without OS benefit after weighing against its potential 10–15% severe toxicity risks, 35% long-term toxicity risks, and diminished quality of life risks, respectively. Patients with stage IIB/C and stage III/IV melanoma were willing to tolerate adjuvant therapy with no more than 21% and 22% risk of significant side effects, respectively, while gaining minimum DFS absolute benefits of 16% over 2 years and 30% over 5 years, respectively.

**Conclusions**

Our study highlighted a diverse patients’ perceptions of meaningful study endpoints, magnitude of treatment benefits, and treatment-related risks tolerance. These insights underscore the importance of integrating patient preferences into trial designs and treatment discussions to ensure patient-centered care in the management of advanced melanoma.

## 8. Oral Presentation|08_CAMO_2024

Award Recipient

**Lost Chances: The Systematic Exclusion of Patients with Brain Metastasis in Early-Phase Oncology Trials ^†^**

Italo Fernandes ^1^, Carlos A. Carmona-Gonzalez ^1^, Shonekaa Suthaaharan ^2^, Serena Sandhu ^3^, Ines Menjak ^1^, Martin Smoragiewicz ^1^ and Katarzyna J. Jerzak ^1^

1. Sunnybrook Health Sciences Centre, Toronto, ON, Canada
2. McMaster University, Hamilton, ON, Canada
3. Sunnybrook Research Institute, Toronto, ON, Canada

^†^  Only the abstract. This was presented as a poster in the ASCO-SNO conference in August 2024. But the search was updated and presented in Ottawa.

Historically, cancer trials have often excluded patients with brain metastases (BrM). This limits our understanding of CNS-specific drug efficacy. We aimed to determine the proportion of phase I clinical trials for patients with solid tumors that exclude patients with BrM.

**Methods**

In March 2023, a systematic search of the clinicaltrials.gov website was performed. The results are described as frequencies and compared using the Pearson’s chi-square or Cochran-Armitage tests.

**Results**

In total, 1714 phase I trials were identified and 990 met the inclusion criteria. Most trials spanned several tumor types (*n* = 659, 66.6%) and were registered between 2000 and 2023 (*n* = 884, 89.3%). Trials were mainly conducted in Asia (*n* = 95, 9.6%), Europe (*n* = 112, 11.3%), and North America (*n* = 619, 62.5%). Categories of investigational agents were: targeted therapy (*n* = 496, 50.1%), cytotoxic chemotherapy (*n* = 118, 11.9%), immunotherapy (*n* = 73, 7.37%); the most common combinations included chemotherapy plus targeted therapy (*n* = 265, 26.8%) and chemotherapy plus immunotherapy (*n* = 14, 1.4%). Patients with BrM were included in 25.1% (*n* = 248) trials, included under certain conditions in 53.6% (*n* = 521), and excluded in the remaining 22.3% (*n* = 221) of trials. Recent trials (2015–2019) were less likely to exclude patients with BrM compared to trials conducted between 2000 and 2004 (18% vs. 38%, *p* = 0.003). Exclusion of patients with BrM was less common in trials focusing on melanoma (16%), breast (15%) and lung (14%) cancer. Trials involving the use of cytotoxic chemotherapy were significantly more likely to exclude patients with BrM (*n* = 58, 49%) than those employing targeted therapy (*n* = 110, 22%) or immunotherapy (*n* = 15, 21%) (*p* = 0.016). Trial location and stage of disease were not associated with exclusion of patients with BrM.

**Conclusions**

Recent phase I trials, particularly those evaluating the use of targeted therapy and/or immunotherapy, have a low rate of exclusion of patients with BrM compared to previous cytotoxic trials. However, many opportunities to test drugs with potential CNS efficacy are still lost.

## 9. Oral Presentation|09_CAMO_2024

**HER2-Low Status Is Associated with Time to Development of Brain Metastases Among Patients with Breast Cancer: A Retrospective Cohort Study †**

Kevin Yijun Fan ^1,2^, Rania Chehade ^2^, Italo Fernandes ^2^, Veronika Moravan ^3^ and Katarzyna Joanna Jerzak ^1,2^

1. University of Toronto, Toronto, ON, Canada
2. Sunnybrook Odette Cancer Centre, Toronto, ON, Canada
3. VM Stats, Toronto, ON, Canada

^†^  Abstract published 2024 August (Neuro-Oncology Advances) following presentation at international conference (ASCO/SNO CNS metastases conference). https://pmc.ncbi.nlm.nih.gov/articles/PMC11296805/. Full paper not yet published.

**Objective**

Brain metastases (BrM) develop in a high proportion of patients with breast cancer. We aimed to investigate the association between HER2 status (HER2-0, HER2-low, HER2+) and time to BrM development in patients treated for breast cancer BrM.

**Methods**

We investigated a cohort of 188 women with metastatic breast cancer (MBC) treated for BrM at Sunnybrook Odette Cancer Centre from 2008 to 2018 for whom HER2 status was available in either the primary breast cancer (PBC) or metastatic site. We investigated the following: (1) association between HER2 status of the PBC (when available) and time from PBC to BrM diagnosis (PBC-TTBM), and (2) association between HER2 status of either PBC or metastatic site (the higher level of HER2 expression was utilised when >1 sample was available) and time from MBC to BrM diagnosis (MBC-TTBM).

**Results**

Median age was 50 years and 54% of patients had >1 BrM. A total of 40 patients had triple-negative breast cancer (TNBC), 77 had HER2+ disease, and 70 had HR+ (hormone receptor+)/HER2- disease. Among 129 patients for whom PBC HER2 status was available, the median PBC-TTBM was 44 months. HER2-low (*n* = 29) status was associated with shorter PBC-TTBM compared to HER2-0 (*n* = 60) and HER2+ (*n* = 40) status (33 vs. 50 vs. 52 months, *p* = 0.045). Among patients with HR+/HER2- disease, HER2-low status (*n* = 21) was associated with shorter PBC-TTBM compared to HER2-0 status (*n* = 29) (34 vs. 95 months, *p* < 0.001). Among patients with TNBC, PBC-TTBM was similar among patients with HER2-low (*n* = 8) and HER2-0 (*n* = 31) status (24 vs. 27 months, *p* = 0.79). In all patients, median MBC-TTBM was 21 months. MBC-TTBM was similar among patients with HER2-0 (*n* = 58), HER2-low (*n* = 53) and HER2+ (*n* = 77) disease (15 vs. 19 vs. 22 months, *p* = 0.75); no differences were observed within the HR+/HER2- and TNBC subgroups.

**Conclusions**

HER2-low status is associated with shorter PBC-TTBM, particularly among patients with HR+/HER2- disease.

## 10. Oral Presentation|10_CAMO_2024

**Financial Conflicts of Interest Among Phase III Randomized Controlled Trials (RCTS) Evaluating Immune Checkpoint Inhibitors for Genito-Urinary Malignancies**

Karam Elsolh ^1^, Sabrina Sikka ^1^ and Sebastien J. Hotte ^1^

1. Juravinski Cancer Centre, Department of Medical Oncology, McMaster University, Hamilton, ON, Canada

**Introduction and Objective**

Immune checkpoint inhibitors (ICIs) are high-grossing medications in oncology. Similarly high-grossing medications in other fields have been associated with financial conflicts of interest (FCOIs) among authors of research studies. The aim of this study is to determine the prevalence of FCOIs among authors of RCTs of ICIs in common GU malignancies.

**Methods**

We ran a systematic search on EMBASE, Ovid, and Cochrane Library for phase III RCTs investigating ICIs in common GU malignancies (bladder cancer, prostate cancer, and renal cell carcinoma). The primary endpoint was prevalence of relevant FCOIs among authors (defined as payments from pharmaceutical manufacturers of the investigated ICIs). FCOIs were stratified according to disclosure status and payment type (general, research, and/or stock/ownership). Undisclosed FCOIs to US-based physicians were identified using the Centre for Medicare and Medicaid Services (CMS) Open Payments Database.

**Results**

Twenty-five RCTs were identified. FCOIs were found in 80.5% of author entries (528 of 656). Seventy-two percent disclosed FCOIs from the ICI manufacturer(s) (424 of 656). Among authors with CMS Open Payments accounts, 38% had additional undisclosed FCOIs (54 of 142). The most common FCOIs were general payments (438, 66.8%), followed by research payments (222, 33.8%) and stock/ownership (89, 13.6%). The median dollar value per author of FCOIs was $11,959 (IQR: $4021–$37,408) in general payments, $1926 (IQR: $1017–$4433) in research payments, and $647,898.31 (IQR: $253,983–$1,246,653) in associated research funding. Median conflict dollar value (excluding study-related research funding) was $13,790 (IQR: $4133 to $36,179).

**Conclusions**

FCOIs from ICI manufacturers were highly prevalent among authors of phase III RCTs. In addition, there was a high burden of undisclosed FCOIs among authorship. Increased disclosure in FCOIs is needed to ensure greater transparency in research investigating ICIs, and we recommend stronger adherence to standardized reporting guidelines for conflict payments.

## 11. Oral Presentation|11_CAMO_2024

**Predictors of Short-Term Death and Long-Term Survival in Advanced Hepatocellular Carcinoma (HCC) Treated with Contemporary Tyrosine Kinase Inhibitors or Immunotherapy: A Multicentre Analysis from the HCC-CHORD Consortium †**

Pooya Dibajnia ^1^, Mark Freeman ^2^, Tharani Krishnan ^3^, Cha Len Lee ^4^, Ravi Ramjeesingh ^5^, Joao Paulo Solar Vasconcelos ^3^, Hanna Lyubetska ^6^, Esther Provost ^1^, Jessica Li ^1^, Philip Q. Ding ^2^, Howard J Lim ^3^, Jennifer J Knox ^4^, Vallerie Lynn Gordon ^6^, Winson Y. Cheung ^2^, Vincent C. Tam ^2^ and Brandon M. Meyers ^1^

1. Juravinski Cancer Centre, McMaster University, Hamilton, ON, Canada
2. Tom Baker Cancer Centre, University of Calgary, Calgary, AB, Canada
3. BC Cancer–Vancouver, University of British Columbia, Vancouver, BC, Canada
4. Princess Margaret Cancer Centre, University Health Network, University of Toronto, Toronto, ON, Canada
5. Nova Scotia Cancer Center, Dalhousie University, Nova Scotia, NS, Canada
6. CancerCare Manitoba, University of Manitoba, Winnipeg, MB, Canada

^†^  This project was presented at ASCO 2024, and the abstract was published in the *Journal of Clinical Oncology* as part of the conference proceedings. A full paper has NOT been published. Please see below for link to the published abstract: https://ascopubs.org/doi/10.1200/JCO.2024.42.16_suppl.4098.

**Objective**

To identify predictors of short-term death (STD) and long-term survival (LTS) for HCC patients treated with TKIs or immunotherapy combinations.

**Methods**

Retrospective review of patients with advanced HCC who received a tyrosine kinase inhibitor (TKI) or immunotherapy combinations was conducted between Aug 2018 to Aug 2022 in British Columbia, Alberta, Nova Scotia, Manitoba, as well as Juravinski and Princess Margaret Cancer Centres in Ontario. Prior locoregional therapies were permitted. Variables examined were: Barcelona Clinic Liver Cancer (BCLC) stage, Child-Pugh class, albumin-bilirubin (ALBI) grade, alpha fetoprotein (AFP) level, microvascular invasion (MVI), and distant metastases. Multivariate logistic regression was used to determine independent predictors of STD (patient survival < 6 months vs. others) and LTS (patient survival > 18 months vs. others).

**Results**

520 patients with the following baseline characteristics were included: median age 66, 84.6% male, 87.4% ECOG performance status 0–1, 69.7% BCLC stage C, and 88.7% Child-Pugh A. First-line treatments were primarily: lenvatinib (57.3%), sorafenib (8.1%), and atezolizumab/bevacizumab (31.7%). Median progression-free survival was 7.4 months (95%CI, 6.2 to 8.4), and overall survival was 17.1 months (95%CI, 15.1 to 18.8). Independent predictors of STD were (odds ratio, 95%CI): ALBI grade (2.26, 1.29–4.07), MVI (2.13, 1.14–4.00), and distant metastases (2.14, 1.15–4.06). These three factors were also predictive for LTS: ALBI grade (0.45, 0.28–0.73), MVI (0.49, 0.26–0.89), and distant metastases (0.36, 0.20–0.65). Child-Pugh class, AFP, and BCLC stage were not predictive of STD nor LTS.

**Conclusions**

This study supports higher ALBI grade, MVI and distant metastases as predictors of STD, while lower ALBI grade, absence of MVI and lack of distant metastases were predictive of LTS in HCC patients treated with contemporary TKIs or immunotherapy combination regimens. These predictors may help guide treatment decisions in patient populations underrepresented in clinical trials, including Child-Pugh B7 patients often excluded from trials.

## 12. Oral Presentation|12_CAMO_2024

**Adjuvant Endocrine Therapy in Premenopausal Women with High-Risk Hormone Receptor-Positive Breast Cancer: A Survey of Practice Variations Amongst Canadian Medical Oncologists**

Sarah Devereaux ^1^, Jadyn Normore ^2^ and Erin Powell ^3^

1. Department of Medicine, Memorial University of Newfoundland, St. John’s, NL, Canda
2. Faculty of Medicine, Memorial University of Newfoundland, St. John’s, NL, Canada
3. Department of Medical Oncology, Memorial University of Newfoundland, St. John’s, NL, Canada

**Objective**

The goal of this survey-based study was to assess how Canadian Medical Oncologists have navigated the challenges associated with real-world implementation of combination ovarian function suppression (OFS) and aromatase inhibition (AI) in the treatment of premenopausal women with high-risk, hormone receptor-positive breast cancer.

**Methods**

Following ethics board approval, our 18-question electronic survey was sent to Canadian medical oncologists who specialize in breast cancer. The survey questions were developed based on a literature review and anecdotal challenges related to OFS and AI faced by patients and providers at our Cancer Centre in St. John’s, NL. We performed a quantitative data analysis to determine the most frequently chosen responses, in an attempt to define a national consensus on each topic. We also performed a qualitative analysis of free text responses, with a focus on key themes.

**Results**

We received 21 survey responses from participants across eight Canadian provinces. Our results revealed a significant variation in patient preferences and clinician approach to the several practical challenges associated with the use of OFS and AI. There were only four questions where >75% of respondents were in agreement. Significant variability was identified amongst decisions regarding sequencing of treatment, patient-directed premature cessation of treatment, and more. Amongst free text responses, prominent themes included provider uncertainty around several aspects of this regimen, as well as treatment toxicity and associated poor patient tolerance.

**Conclusions**

This study highlights a lack of consensus amongst providers who treat premenopausal women with high-risk, hormone receptor-positive breast cancer, and thus the need for clearer guidance in this area. With the overall goal of standardization of care across our country, a national guideline document addressing these points of uncertainty in practice is needed. Additionally, further research to explore methods of reducing treatment toxicity would likely improve treatment adherence in this area.

## 13. Poster Presentation|13_CAMO_2024

Award Recipient

**Impact of a National Virtual Oncology Course on Medical Student Competency and Professional Identity Development**

Aaron Dou ^1^, Joy Du ^2^, Joanne Alfieri ^3^, Jennifer Croke ^4^, T.P.L Nghiem ^5^, Kimberly DeVries ^5^, Sharlene Gill ^6^ and Paris-Ann Ingledew ^7^

1. Department of Medicine, University of Toronto, Toronto, ON, Canada
2. Department of Medicine, McMaster University, Hamilton, ON, Canada
3. Division of Radiation Oncology, McGill University Health Centre, Montreal, QC, Canada
4. Department of Radiation Oncology, University of Toronto, Toronto, ON, Canada
5. Department of Clinical Research, Data & Analytics, BC Cancer, Vancouver, BC, Canada
6. Division of Medical Oncology, University of British Columbia, Vancouver, BC, Canada
7. Division of Radiation Oncology, University of British Columbia, Vancouver, BC, Canada

**Purpose**

Past surveys have identified that Canadian medical learners of all levels perceive their oncology curricula and training to be inadequate. Moreover, oncology remains highly represented on the Medical Council of Canada Qualifying Examination (MCCQE) Part I, accounting for 30% of clinical objectives. To address this gap, we systematically developed and implemented the Oncology National Course for Advocacy, Research, and Education (ONCARE) and evaluated its impact on oncology competency and professional identity development.

**Materials and Methods**

ONCARE was designed utilizing Kern’s Six-Step Model for Curriculum Development, including a literature review and targeted needs assessment, and informed by the Canadian Oncology Goals and Objectives for Medical Students. Components of ONCARE included lectures, career panels, and a mentorship program. Data were collected from students through surveys and reflective essays, and analyzed using a mixed-method approach involving descriptive analysis as well as qualitative thematic analysis with an interpretive research paradigm. Statistical analysis was conducted using two-tailed paired *t*-tests or McNemar’s paired Chi-square test with α = 0.01. Kirkpatrick’s framework was applied to evaluate course impact.

**Results**

245 students representing 14 medical schools across Canada enrolled in ONCARE between October 2023–February 2024, of which 207 (84%) were preclerkship students. All students completed the pre-course survey and 118 (48%) completed the post-course survey. Prior to ONCARE, 54% of students (*n* = 123) felt they received an inadequate amount of oncology teaching. Paired analysis of students revealed that ONCARE improved students’ perception of their oncology knowledge relative to other medical school topics (*p* < 0.001) and confidence with communication skills in oncology (e.g., breaking bad news) (*p* < 0.001). Baseline interest in oncology was high (mea*n* = 3.9/5) and did not significantly change after the course (*p* = 0.89). Qualitative analysis of reflective essays has identified several themes pertaining to professional identity development, including: (1) impact of mentorship on specialty selection; (2) exposure to new oncology disciplines, and (3) importance of lifestyle in specialty selection. Factors that drew students towards oncology included personal experiences with cancer and opportunities for longitudinal patient care, whereas fear of emotional burnout was cited as a deterrent. The rapidly advancing research and treatment options in oncology was both a facilitator and a deterrent for students considering oncology.

**Conclusions**

Analysis of ONCARE data has shown that a virtual educational intervention can influence professional identity formation and self-perceived oncology competency for medical students. Future work will assess ONCARE’s longitudinal impact and utilize our findings to inform oncology curriculum development through partnership with Canadian medical schools.

## 14. Poster Presentation|14_CAMO_2024

**Provincial Treatment Practices & Toxicities in Patients with Early Stage HER2 Positive Breast Cancer**

Lyubetska, H. ^1,2^, Kim, C.A. ^1,2^, Jassal, D. ^1,3^, Bucher, O. ^4^, Feely, A. ^4^ and Grenier, D ^1,2^

1. Department of Internal Medicine, University of Manitoba, Winnipeg, MB, Canada
2. Section of Hematology/Oncology, University of Manitoba, Winnipeg, MB, Canada
3. Section of Cardiology, University of Manitoba, Winnipeg, MB, Canada
4. Department of Epidemiology and Cancer Registry, CancerCare Manitoba, Winnipeg, MB, Canada

**Objective**

To describe the treatment patterns, cardiac adverse effects and outcomes of Manitoba patients with early stage HER2 positive breast cancer, treated with trastuzumab and an anthracycline versus a non-anthracycline chemotherapy backbone.

**Methods**

A retrospective cohort study of patients with HER2 positive early-stage breast cancer treated with adjuvant trastuzumab and chemotherapy between 2010 and 2014. Using the Manitoba Cancer Registry, a total of 346 patients were identified, 63 of whom were excluded due to limited information. Baseline patient and treatment characteristics were recorded, including age, stage, comorbidities, cardiovascular risk factors, and chemotherapy regimen received (anthracycline vs. no anthracycline). We also report survival according to anthracycline versus non-anthracycline chemotherapy. The frequency and type of cardiac monitoring, as well as rates of cardiotoxicity, were examined.

**Results**

Of 283 patients, 190 received an anthracycline regimen and 93 received a non-anthracycline regimen combined with trastuzumab. Patients who received anthracycline-based regimens were younger, had high-grade disease (*p* = <0.0001) and more advanced stage disease (*p* = <0.0001), and had a greater number of lymph nodes involved (*p* = <0.001). A higher proportion of those who received a non-anthracycline regimen had cardiac risk factors, primarily hypertension (*p* = 0.0018). Numerically, patients treated with anthracyclines developed more adverse cardiac events, but this did not demonstrate statistical significance. There was also no difference in survival between the two groups. The majority of patients in both groups had an MUGA scan for cardiac monitoring every three months while on treatment.

**Conclusions**

The optimal chemotherapy partner for trastuzumab in the adjuvant setting remains unclear. Local data suggest that patient and disease characteristics may influence who does or does not receive anthracycline-based treatment. Despite these differences, no survival benefit was seen for those who received anthracycline.

## 15. Poster Presentation|15_CAMO_2024

**Evaluating Alternative Dosing Regimens of Consolidative Durvalumab in Patients with Stage III NSCLC Treated with Chemoradiotherapy: An Observational Cohort Study**

Jonathan Moroniti ^1^, Gabrielle Pundaky ^1^, Sarah Ma ^1,3^, Eric McArthur ^2,3^, Nawar Tarafdar ^1^, Robin Sachdeva ^4^, Saritha Surapaneni ^2,3^ and Sara Kuruvilla ^1,2,3^

1. Schulich School of Medicine and Dentistry, Western University, London, ON, Canada
2. Department of Oncology, London Regional Cancer Program, London Health Sciences Center, 800 Commissioners Road East, London, ON, Canada
3. Lawson Health Research Institute, 750 Base Line Rd E, London, ON, Canada
4. Precision for Medicine, 800 René-Lévesque Blvd. West, 26th Floor. Montréal, QC, Canada

**Objective**

Durvalumab 10 mg/kg every two weeks for one year after chemoradiation improves survival in unresectable Stage III non-small-cell lung cancer (NSCLC). Cancer Care Ontario approved a 20 mg/kg four-weekly regimen in response to the COVID-19 pandemic. Southwestern Ontario serves a demographically distinct population, and uncertainty remains regarding the impact of this modification. This study aimed to compare the effectiveness and tolerability of these two regimens.

**Methods**

We reviewed the medical records of consecutive adult patients diagnosed with Stage III NSCLC from 1 January 2018 to 4 May 2023 who were treated with chemoradiation at the London Health Sciences Centre, Ontario, Canada. Information collected included patient demographics, durvalumab dosing frequency, progression pattern, survival outcomes, and adverse events. Patients were categorized according to durvalumab dosing schedule. Descriptive statistics were applied to demographic, treatment-related, and clinico-pathologic characteristics. Overall survival and real-world progression free survival analyses were conducted using Kaplan–Meier curves and Cox proportional hazard models. The research protocol was reviewed and approved by the Institutional Research Ethics Board Review.

**Results**

A total of 196 patients were included. The median age was 70 years old, 53% were male, most were current or former smokers (>90%), and the majority had an ECOG-PS of 0–1 (89%). A total of 45% of patients had adenocarcinoma, and 29% had squamous cell carcinoma. In total, 66% of patients received carboplatin, while 36% received cisplatin. Following chemoradiotherapy, 27% of patients received the two-week durvalumab regimen (Durvalumab-Q2w), 63% received the four-week regimen (Durvalumab-Q4w), and 10% did not receive durvalumab. There was no significant difference in the risk of death (HR: 0.70, 95%CI: 0.42–1.14; *p* = 0.15) or progression (HR: 0.77, 95%CI: 0.52–1.14; *p* = 0.19) between the two dosing groups. Toxicity profiles were similar between the two groups.

**Conclusions**

This study identified the comparable effectiveness and tolerability of consolidative durvalumab administered every four weeks to every two weeks, supporting further investigation of its use post-pandemic.

## 16. Poster Presentation|16_CAMO_2024

**Enhancing the Patient Journey to Clinical Trial Enrollment with Navigation to Optimize Accrual: A Pilot Study for a Pragmatic Multicentre, Stepped Wedge, Cluster Randomized Control Trial (The CTN Pilot Trial) ^†^**

Emmanuel Akingbade ^1^, Mahmoud Hossami ^1^, and Rhonda Abdel-Nabi ^1^, Farwa Zaib ^2^, Kayla Touma ^1^, Renee Nassar ^2^, Sanghyuk Claire Rim ^2^, Milica Paunic ^1^, Olla Hilal ^2^, Pratham Gupta ^2^, Roaa Hirmiz ^3^, Michael Touma ^4^, Govana Sadik ^1^, Depen Sharma ^1,^ Swati Kalia ^2^, Rija Fatima ^1^, Anthony Luginaah ^2^, Ibrahim Mohamed ^2^, Rong Luo ^1^, Megan Delisle ^5,6^, Caroline Hamm ^1,2,3^

1. University of Windsor, Windsor, ON, Canada
2. Western University, London, ON, Canada
3. Clinical Trials Navigator Inc.
4. Griffith University in Gold Coast, Queensland, Australia
5. Department of Surgery, University of Manitoba, Winnipeg, MB, Canada
6. Paul Albrechtsen CancerCare Manitoba Research Institute, Winnipeg, MB, Canada

^†^  This abstract has been published in the Journal of Clinical Oncology. *Journal of Clinical Oncology,* Volume 42, Number 16_suppl, https://doi.org/10.1200/JCO.2024.42.16_suppl.11093.

**Background**

Clinical trials are essential to the advancement of clinical therapies, yet accrual rates remain disappointing. Multiple challenges lead to less than 5% of cancer patients enrolled onto clinical trials. The Clinical Trials Navigator (CTN) program was established to assist patients and healthcare professionals identify appropriate clinical trials for patients.

**Methods**

Between March 2019 and January 2024, a novel navigator-assisted clinical trials search program was offered to Canadian patients. Three non-medical navigators were trained to receive referrals, review medical information, and search five different clinical trial search engines. Eligibility criteria were scrutinized. A second review of the clinical trial list was conducted by two physicians. The final curated list of clinical trials was provided to patients and their oncologist.

**Results**

A total of 373 patients were referred to the CTN program during the study period. A unique clinical trial search was performed for each patient yielding a median of only one potentially eligible trial per patient. Clinical trial enrolment occurred in 3.2% of patients in our database which translates to a 19% rate of successful enrolment of those referred to a trial by the CTN. Most patients (78%) were referred to clinical trial sites that conducted more than 100 clinical trials at any time.

Compared to the Canadian cancer statistics, lung, lymphoma, pancreatic and brain cancers were overrepresented in referrals to the CTN program while prostate cancer was underrepresented. Type of cancer played a significant role in the likelihood of a successful referral (*p* < 0.01). Lung cancer was the most frequently reported cancer that resulted in referrals and breast cancer showed a lower frequency of referrals. The cancer type, stage and number of lines of prior therapy were not significantly associated with the patient enrollment onto a clinical trial. An increase in survival of referred patients from last analysis from 3.0 months to 5.3 months.

**Conclusions**

The CTN program is a successful tool to identify clinical trials for cancer patients and can improve clinical trial accrual, as almost one fifth of patients (19%) who were referred to a clinical trial were enrolled. Ongoing iterative changes to the program to improve these metrics are underway and efforts to improve implementation of the CTN program across Canada are ongoing.

## 17. Poster Presentation|17_CAMO_2024

**A Real-World Analysis of Total Neoadjuvant Therapy and Other Regimens for Locally-Advanced Rectal Cancer Treatment**

Brian Shih ^1^, Beatrice TB Preti ^2,3^, Wei C Wang ^1^, Michael Merrick ^1^, Eric McArthur ^4^ and Elena Tsvetkova ^2^

1. Schulich School of Medicine & Dentistry, Western University, London, ON, Canada
2. Division of Medical Oncology, Schulich School of Medicine & Dentistry, Western University, London, ON, Canada
3. Division of Medical Oncology, Emory School of Medicine, Emory University, Atlanta, GA, USA
4. London Health Sciences Centre, London, ON, Canada

**Objective**

To assess the efficacy, toxicities, and other patient-relevant factors of individuals receiving total neoadjuvant therapy (TNT) and non-TNT for locally-advanced rectal cancer (LARC).

**Methods**

We conducted a retrospective analysis of all patients treated for LARC at the London Regional Cancer Program (LRCP) between 1 July 2019 and 14 July 2022. In June 2020, TNT was employed as a new treatment approach. In total, 124 variables were collected, including patient demographics, treatment type, adverse events, response to treatment, surgery versus watchful waiting, and survival. Data were analysed using descriptive statistics.

**Results**

A total of 617 potential patients were identified by medical records, of which 290 were included in the final analysis. Reasons for exclusion were treatment at external sites, metastatic disease at presentation, or treatment outside of date parameters. Of patients treated, 64.5% (*n* = 187) were male. Median patient age was 67 years, with 51.0% (*n* = 148) having stage III disease. Sixty-four patients (22.1%) received TNT, 109 patients (37.6%) received non-TNT neoadjuvant treatment, and 69 patients (23.8%) received non-TNT neoadjuvant plus adjuvant therapy. Differences in treatment response categories between TNT and non-TNT were significant (*p* = 0.012). For patients receiving TNT, 43.8% (*n* = 28) showed complete response, 31.3% (*n* = 20) showed partial response, and 9.4% (*n* = 6) showed disease progression. In the non-TNT cohort, 23.9% (*n* = 54) showed complete response, 34.1% (*n* = 77) showed partial response, and 8.4% (*n* = 18) showed disease progression. Following neoadjuvant therapy, TNT and non-TNT cohorts had 60.9% (*n* = 39) and 62.4% (*n* = 141) proceed to surgical intervention, respectively.

**Conclusions**

Individuals with LARC demonstrate significantly higher rates of response to treatment with TNT compared to non-TNT approaches. The standardization of TNT as an approach for LARC may improve both patient outcomes and rates of organ preservation.

## 18. Poster Presentation|18_CAMO_2024

**Real-World Outcomes Associated with Immune Checkpoint Inhibitors in Recurrent or Metastatic Head and Neck Squamous Cell Cancer (R/M HNSCC) Patients**

Haya Abuzuluf ^1^, Wilma Hopman ^2^ and Andrea S. Fung ^3^

1. Department of Medicine, Queen’s University, Kingston, ON, Canada
2. Department of Public Health Sciences, Queen’s University, ON, Canada
3. Department of Oncology, Queen’s University, Kingston, ON, Canada

**Objective**

Real-world data on palliative immune checkpoint inhibitor (ICI) therapy in R/M HNSCC are limited. This study investigates treatment outcomes associated with ICIs in R/M HNSCC patients.

**Methods**

A retrospective analysis of R/M HNSCC patients treated with first-line palliative ICI at the Cancer Centre of Southeastern Ontario (2016–2023). Data were summarized using descriptive statistics. Overall survival (OS) and progression-free survival (PFS) were evaluated in patients treated with ICI alone (I) or in combination with chemotherapy (C/I) using the Kaplan–Meier method.

**Results**

Out of 34 patients, the median age was 63 years, 26 (76.5%) were male, 28 (82.4%) ECOG 0–1, 18 (52.9%) current smokers, and 25 (73.5%) current alcohol consumption. Twenty patients had oropharyngeal (OP) cancer (58.8%), 7 (20.6%) laryngeal cancer, and 4 (11.8%) oral cavity cancer. In OP patients, 72.2% were p16-positive. PD-L1 status was not routinely tested. Twenty-three (67.6%) patients had recurrent metastatic disease, 17.6% de novo metastatic and 14.7% locoregional recurrence. Most had prior local therapy: 15 (44.1%) chemoradiation, 9 (26.4%) surgery, 4 (11.8%) radiation alone. For first-line palliative systemic therapy, 25 (73.5%) received I alone and 9 (26.5%) had C/I. The most common reason for treatment discontinuation was progression (73.5%). Only 3 patients (8.8%) had immunotoxicity. Median OS was 10.8 months (95%CI: 7.25–14.27) in C/I group and 7.1 months (95%CI: 3.41–10.76) in I alone group (*p* = 0.72), while median PFS was 6.5 months (95%CI: 5.36–7.56) vs. 5.2 months (95%CI: 4.59–5.88), respectively (*p* = 0.46). In OP patients, mOS was similar despite p16 status: 10.8 months (95%CI: 7.60–13.92) in p16-positive vs. 8.5 months (95%CI: 0.73–16.23) in p16-negative group (*p* = 0.864).

**Conclusions**

There was no significant difference in survival outcomes between the different treatment regimens, or by p16 status in OP patients. Our results are limited by a small sample size and should be further validated in larger cohorts.

## 19. Poster Presentation|19_CAMO_2024

**Clinical and Laboratory Characteristics That Influence Survival of Men with Metastatic Castrate Resistant Prostate Cancer Treated with Radium-223**

Lorin Dodbiba ^1,2^, Gregory Pond ^1^ and Sebastien J. Hotte ^2^

1. Health Research Methodology, McMaster University, Hamilton, ON, Canada
2. Division of Medical Oncology, Juravinski Cancer Centre, Hamilton, ON, Canada

**Objective**

Determine which laboratory and clinical factors are predictive of better survival outcomes in patients treated with Radium-223. Develop tools or criteria to better select patients who may benefit from Radium-223 treatment while minimizing potential toxicities.

**Methods**

Pre-treatment laboratory and clinical data were collected via the Institute for Clinical Evaluative Sciences (ICES) from patients with prostate adenocarcinoma who received at least one Radium-223 treatment. Physician billing codes were used to identify Radium-223, prior chemotherapy, and radiation, while drug identification numbers (DINs) were used to identify androgen receptor pathway inhibitors (ARPIs) and bone-modifying agents (denosumab or zoledronate). Cox proportional hazard models were used to explore which factors were prognostic for survival.

**Results**

Between 3 March 2014 and 31 December 2023, 1588 patients with prostate adenocarcinoma received Radium-223 in Ontario. At time of treatment, median age was 68 and median time from diagnosis was 4.6 years. Almost half had stage IV disease at diagnosis (45.8% or *n* = 728), while 23.8% (377) were diagnosed at an early stage (I + II). Prior use of ARPIs was seen in 27.6% (438) of patients, while 25.3% (401) had prior use of a bone-modifying agent. Median survival from first Radium-223 treatment was 12.3 (95% confidence interval (95%CI) = 11.3–13.3) months. In a multivariable model, higher hemoglobin (hazard ratio (HR) = 0.80, 95%CI = 0.76–0.83), calcium (HR = 0.22, 95%CI = 0.15–0.32) and platelets (HR = 0.88, 95%CI = 0.80–0.96) at Radium-223 treatment start were significantly associated with longer survival, while prior ARPI use (HR = 1.29, 95%CI = 1.11–1.51) and higher neutrophils (HR = 1.12, 95%CI = 1.09–1.15) were associated with shorter survival. Excluding laboratory values, >5 years between diagnosis and Radium-223 (HR = 1.28, 95%CI = 1.12–1.46) and prior use of ARPIs (HR = 1.23, 95%CI = 1.08–1.40) were negatively prognostic, while stage I at diagnosis (HR = 0.38, 95%CI = 0.21–0.68) was associated with better survival.

**Conclusions**

These results can help inform patient selection regarding who might benefit from Radium-223 treatment. Further research to create a selection model with formal external validation is warranted.

## 20. Poster Presentation|20_CAMO_2024

**Primary Cancer Site and Extracranial Progression Are Associated with Development of Brain Metastases in Patients Treated with Stereotactic Body Radiotherapy for Oligometastatic Solid Tumours ^†^**

Kevin Yijun Fan ^1,2,‡^, Katarzyna Joanna Jerzak ^1,2,‡^, Sudhir Kumar ^2^, Veronika Moravan ^3^, Badr Id Said ^1,2^, Sunit Das ^2^, Alexander V. Louie ^1,2^, Hany Soliman ^1,2^, Arjun Sahgal ^1,2^ and Hanbo Chen ^1,2^

1. University of Toronto, Toronto, ON, Canada
2. Sunnybrook Odette Cancer Centre, Toronto, ON, Canada
3. VM Stats, Toronto, ON, Canada

^†^  Abstract published 2024 August (Neuro-Oncology Advances) following presentation at international conference (ASCO/SNO CNS metastases conference). https://pmc.ncbi.nlm.nih.gov/articles/PMC11296792/; Full paper published (*Journal of Neuro-Oncology*) 2025 January, https://pubmed.ncbi.nlm.nih.gov/39365544/.
^‡^  KYF and KJJ contributed equally to this work.

**Objective**

Patients with oligometastatic disease (OMD) may achieve long-term control with stereotactic body radiation therapy (SBRT). However, those who develop brain metastases (BrM) often have poor outcomes. We aimed to investigate variables associated with development of BrM in this population.

**Methods**

Patients with ≤5 extracranial metastases from solid tumours treated with SBRT from 2008 to 2016 at Sunnybrook Odette Cancer Centre were included. We investigated the association between covariates and CIBrM (cumulative incidence of BrM) using Fine-Gray analysis with death as a competing risk, and progression-free survival (PFS) and overall survival (OS) using Cox regression. We investigated the association between widespread extracranial progression (≥5 sites) and CIBrM using a time-based conditional competing-risks analysis.

**Results**

Among 404 patients, the most common primary sites were lung (32%), colorectal (26%), prostate (13%), breast (10%) and kidney (7%). Median follow-up was 49 months. Median PFS was 25 months and median OS was 70 months. A total of 58 patients developed BrM, and 5-year CIBrM was 16%. The 5-year CIBrM was highest among patients with breast cancer (22%), lung cancer (15%) and kidney cancer (15%), and lowest among those with colorectal cancer (5%) and prostate cancer (0%). On multivariable analysis, primary site was associated with CIBrM (*p* < 0.001), with colorectal cancer (HR 0.34) and prostate cancer (HR 0.00) associated with lower CIBrM compared to lung cancer. Time from primary diagnosis to OMD, number of extracranial metastases, location of metastases, and total planning target volume (PTV) were not associated with CIBrM. Development of widespread extracranial progression within 24 (HR 4.1, *p* = 0.004), 36 (HR 3.7, *p* = 0.013), 48 (HR 5.9 *p* = 0.009) and 60 (HR 23, *p* = 0.017) months of OMD diagnosis was independently associated with higher CIBrM.

**Conclusions**

Primary site and extracranial progression, but not baseline measures of OMD volume and distribution, are associated with the development of BrM in patients with OMD treated with SBRT.

## 21. Poster Presentation|21_CAMO_2024

**Real-World Evaluation of Clinical Outcomes and Long-Term Survivors in Patients with BRAF-V600E Metastatic Colorectal Cancer (MCRC)**

William J Phillips ^1^, Mohammad Al Rehaili ^1^, Horia Marginean ^2^, Tim Asmis ^1,2^, Mike Vickers ^1,2^, B Lo ^2,3^ and Rachel Goodwin ^1,2^

1. Division of Medical Oncology, Department of Medicine, University of Ottawa, Ottawa, ON, Canada
2. The Ottawa Hospital Research Institute, Ottawa, ON, Canada
3. Eastern Ontario Regional Laboratory Association (EORLA)

**Introduction**

The goal of this study is to evaluate the clinicopathologic features and natural history of BRAF-V600E mCRC in the era prior to combination BRAF/EGFR inhibitors.

**Methods**

This is a retrospective study of patients treated at the Ottawa Hospital Cancer Centre with BRAF-V600E mCRC who did not receive combination BRAF/EGFR therapy. Patients diagnosed with colorectal cancer prior to March 31, 2021, were included. Demographic, clinical, pathologic and cancer characteristics were abstracted from electronic medical records. Outcomes of interest included median duration of therapy and overall survival (OS).

**Results**

A total of 71 patients were included with a median age of 69 years. There were 37 (52%) females. Eighteen (25%) patients had right-sided tumors and 19 (27%) were mismatch repair-deficient (dMMR). The most frequent sites of metastasis included liver (51%), peritoneum (49%), distant lymphatics (37%), lung (20%), bone (9%) and brain (6%). Overall, median OS was 12.9 months, with 20 (28%) patients living greater than 2 years. Signet ring histology (HR = 7.27, *p* < 0.001), peritoneal metastasis (HR = 2.29, 0.003), distant lymphatic metastasis (HR = 2.70, *p* < 0.001), brain metastasis (HR = 2.86, *p* < 0.048) and metastatectomy (HR = 0.17, *p* < 0.001) were significant prognostic factors. A total of 46 (65%) patients received first-line systemic therapy, 14 (20%) second-line and 2 (3%) third-line. The median duration of therapy was 8.5 months for first-line, 5.5 months for second-line and 1.5 months for third-line.

**Conclusions**

Real-world data confirm that patients with BRAF-V600E mCRC have poor clinical outcomes with traditional systemic therapies, although we identified a subset who experienced long-term survival (>2 years). This reflects the real-world heterogeneity in clinical outcomes that are only partially explained by clinicopathologic prognostic factors. We are currently investigating the prognostic value of genomic signatures. Additionally, only a minority of patients received second- or third-line systemic treatments, highlighting the importance of future work evaluating the incorporation of targeted therapy in first-line treatment.

## 22. Poster Presentation|22_CAMO_2024

**Does Lack of Access to Mammograms Impact the Proportion of Patients Receiving Neoadjuvant Therapy (NAT) for Breast Cancer?**

Mollenthiel A ^1,2^, Corke L ^2,3^, Morris A ^2^, Willemsma K ^2,4^, Illman C ^2,4^, Levasseur N ^2,4^ and Simmons CE ^2,4^

1. Temple University, Graduate Pre-Medical Program
2. BC Cancer, Vancouver Centre
3. University of Waterloo, Undergraduate Sciences
4. University of British Columbia

**Introduction/Background**

The pandemic shut down mammograms internationally, from March 2020 to September 2020. With no mammograms for 6 months, there was an expected stage shift in more advanced disease being found post-pandemic and a potential increase in the use of NAT. We wanted to assess if this shift of patients diagnosed with stage 2b or higher did occur, by reviewing the population treated with NAT 2 years before and 2 years after the pandemic. From this we were trying to see the impact of COVID-19 on the BC Cancer system and whether the lack of mammograms caused a stage shift in the disease state of breast cancer.

**Methods**

Using a quality assurance database, we created a query on patients who received NAT between 1 January 2018, and 31 December 2022, at the BC Cancer Vancouver Centre. We filtered for pre-treatment stage and receptor subtype. Using descriptive statistics, we calculated the number and percentage per year of NAT cases with a pretreatment stage 2b or higher. We utilized BC Cancer analytics to identify true and expected rates of diagnosis of breast cancer each year provincially to develop comparative proportions of patients receiving NAT each year.

**Results**

The annual rates of breast cancer diagnosis in BC, were 3860 in 2018, rising to 4165 in 2019, then stayed stable in 2020 at 4160. The number of people diagnosed with breast cancer in 2021 was 4255 and 4355 in 2022. Overall, between 2018 and 2022, there was a 12% increase in the diagnosis of BC. From 1 January 2018 to 31 December 2022, 389 patients received NAT at the BC Cancer Vancouver Centre. Between 2018 and 2020, the number of patients receiving NAT increased by 40% and stayed consistent through 2022. Regarding pre-treatment stage, we found a decrease in the proportion of patients with stage 2B or higher disease. In 2018, the percentage of NAT cases stage 2b or higher was 85%, but this fell to 67% in 2019, 67% in 2020, then 63% in 2021 and 55% in 2022. Overall, there was no change in the proportion of each receptor subtype treated each year, except for the luminal B subtype (ER + PR-Her2-). In 2018 and 2019, the luminal B subtype was 4% and 3%. During 2020, this spiked to 15% but decreased back to pre-pandemic levels in 2021 and 2022 at 5% and 4%, respectively.

**Discussion**

Based on the consistent increase in predicted breast cancer diagnosis from the BC analytics model and the absence of mammogram screening during the pandemic, we thought that there would be an increase in the proportion of patients with stage 2b or higher receiving NAT. Our data showed the opposite trend with a decrease in the % of patients receiving NAT with stage 2b or higher. Interestingly, from 2018 to 2022 there was an increase in patients that received NAT. There was also no meaningful change in receptor subtype year to year except for patients that had luminal B receptor subtype. During the pandemic, this spiked to 15% and then returned to pre-pandemic levels of around 4–5%. The reasons for this are unclear.

**Conclusions**

The data suggest that the severity of breast cancer diagnosis was not significantly altered by the lack of access to mammograms. The use of NAT, however, has increased post-pandemic and remained higher. If a reduced frequency of mammograms did not significantly increase the stage of patients receiving NAT, it merits a closer look into the appropriate frequency of mammograms on breast cancer severity.

## 23. Poster Presentation|23_CAMO_2024

**A Multicenter Physician Survey Evaluating Ki-67 Use in Breast Cancer Management in Canada ^†^**

Jennifer Leigh ^1^, Sharon F McGee ^1,2^, Lisa Vandermeer ^2^, Phillip Williams ^3^ and Moira Rushton ^1,2^

1. Division of Medical Oncology, Department of Medicine, The Ottawa Hospital, Ottawa, ON, CanadaCancer Therapeutics Program, Ottawa Hospital Research Institute, Ottawa, ON, Canada
2. Department of Pathology, The Ottawa Hospital, Ottawa, ON, Canada

^†^  The full article has also been published since the conference (was accepted during the conference). Please see DOI: https://doi.org/10.3390/biomedicines12112471.

**Objective**

The use of Ki-67 endocrine therapy (ET) response for treatment decision making in early-stage breast cancer across Canada is unclear; therefore, we surveyed Canadian physicians to explore current practice patterns, perceptions, and perceived barriers to its’ use in practice.

**Methods**

Physicians were invited by email to participate in an anonymous survey using Microsoft Forms and were eligible if they prescribe systemic therapy for breast cancer in Canada. Respondents were asked to describe their usage of Ki-67, perceptions of the evidence surrounding Ki-67 ET response, and interest in future trials using this approach. Responses are reported descriptively.

**Results**

The survey received 48/163 responses (29.4%), 6 of whom were ineligible as they did not prescribe systemic therapy. Majority (97.6%) reported having access to Ki-67 testing upon request. Guiding decisions on adjuvant Abemaciclib was the most common use (97.6%), followed by adjuvant chemotherapy decisions (16.7%), and prognostication (9.52%). Only 19.0% had used Ki-67 response to pre-operative ET in practice; however, 69.0% reported that they would use it if more easily available. Common barriers identified to this approach included lack of awareness by other providers (54.8%), increased resource requirement (54.8%), lack of timely Medical Oncology consultation prior to surgery (52.4%), potential surgical delays (38.1%), and modest or unclear benefit (19.0%). The majority (85.3%) reported that they would participate in future trials using Ki-67 endocrine response, and that the number of patients for whom treatment decisions were changed (95.2%) and cost analysis (42.3%) were important endpoints to include.

**Conclusions**

Despite the widespread availability of Ki-67 testing, few physicians in Canada currently use it to assess endocrine response, predominantly due to logistical and resource constraints. There is a high level of interest in conducting future trials using this strategy which should focus on disease-related outcomes, feasibility, and the financial impact on the public healthcare system.

## Figures and Tables

**Table 1:** Variants.

Tier 1	ATM(5), BRCA2(2), CDK12(5), FANCA(3), NBN(1)
Tier 2	AKT1(2), BRAF(1), CTNNB1(3), GNAS(1), HRAS(1), MED12(1), MET(1), NBN(1), PALB2(1), PIK3CA(4), PIK3R1(7), PMS2(1), PTEN(1), RB1(1), RNF43(2), SETD2(1), SPOP(6), TP53(12)

